# Initial status and 3-month results relating to the use of biodegradable nitride iron stents in children and the evaluation of right ventricular function

**DOI:** 10.3389/fcvm.2022.914370

**Published:** 2022-08-01

**Authors:** Ling Sun, Jun-jie Li, Yu-kai Xu, Yu-mei Xie, Shu-shui Wang, Zhi-wei Zhang

**Affiliations:** Department of Pediatric Cardiology, Guangdong Provincial People’s Hospital, Guangdong Academy of Medical Sciences, Guangdong Cardiovascular Institute, Guangzhou, China

**Keywords:** biodegradable stent, children, nitride iron stents, pulmonary artery stenosis, right ventricular function

## Abstract

**Background:**

Pulmonary artery stenosis is often associated with congenital heart disease. The aim of the study was to evaluate the efficacy and safety of stenting for branch pulmonary artery stenosis using a biodegradable nitride iron stent (IBS^®^ Angel™) and right ventricular systolic and diastolic function.

**Methods:**

From July 2021 to February 2022, a total of 11 cases (ages ranged from 36 to 86 months old) were included in this pre and post-intervention, prospective, cohort and preclinical study. All cases underwent transthoracic echocardiographic (TTE), chest radiography, along with computed tomography (256-slice scanner, multiple-detector) and right heart catheterization. Different types of biodegradable nitride iron stents were implanted. TTE was performed serially 1 day, 1 month and 3 months after the procedure to evaluate the rate of restenosis and right ventricular function.

**Results:**

Stenting was successful in 11 patients. There were no major adverse cardiovascular events related to the device or to the procedure. Blood perfusion in the branch pulmonary artery was improved immediately. At follow-up, there was no significant restenosis that required re-intervention. None of the patients suffered from in-stent thrombosis, vascular embolism, stent displacement or heart failure. Compared with normal values, there were statistical with regards to FAC, E/A and E′/A′. Furthermore, we found that TAPSE correlated significantly with pulsed Doppler S wave (*p* = 0.008) and left ventricular ejection fraction (*p* < 0.01). The early trans-tricuspid inflow velocities E/E′ (tissue doppler at the lateral tricuspid annulus) correlated significantly with E′/A′ (*p* = 0.009). FAC and E′/A′ were statistically different from those prior to stenting (*p* = 0.041 and *p* = 0.035) when tested one month postoperatively. At three months postoperatively, only E/A showed a statistical difference (*p* = 0.015).

**Conclusion:**

Our analysis suggests that biodegradable nitride iron stents are feasible, safe, and effective in children. Some small improvements were observed in right ventricular systolic and diastolic function after successful transcatheter intervention, although change was not statistically significant due to the small sample number. (A clinical Trial to Evaluate the Safety and Efficacy of IBS Angel in Patients With Pulmonary Artery Stenosis (IRIS); NCT04973540).

## Introduction

Pulmonary artery stenosis (PS) is often associated with congenital heart disease (CHD) and a wide range of genetic syndromes, thus leading to elevated right ventricular or main pulmonary artery pressure, arrhythmia, imbalanced lung perfusion, systemic cyanosis, and sudden death (1–3). At present, treatment options include surgery, balloon angioplasty and stent implantation. However, surgery leads to significant trauma and scarring, and it remains a significant challenge to resolve stenosis in the distal branch of the pulmonary artery. Some intermediate-term clinical trials have shown that balloon angioplasty is significantly less effective than stent placement (4). Stent implantation has proven to be a better option and is recommended by the American Heart Association as a class I indication for branch pulmonary artery stenosis (1).

Many different types of stents are available, including open-cell or closed-cell stents, unmounted or pre-mounted stents and covered or bare-metal stents. However, these are not suitable for infants or small children due to vessel restenosis in the later stages of blood vessel growth (5). At present there are no ideal stents that exhibit the advantages of easy implantation into the blood vessels of infants *via* the femoral vein or artery that allow unrestricted vascular growth (6–8). Currently, biodegradable stents are very promising options for the pediatric patients who require stent therapy (9). These stents can provide temporary vascular scaffolding which does not restrict the growth of blood vessels but can withstand the pressure of vasoconstriction and continue to provide support until they are eventually absorbed by human tissues (10, 11). To date, few clinical trials have focused on the application of biodegradable vascular stents in the blood vessels of infants and children. Therefore, we performed a prospective and multicenter clinical trial initiated by Guangdong Provincial People’s Hospital (Guangzhou, China). Before commencing the trial, we carried out a preliminary trial involving eleven cases using biodegradable nitride iron stents.

## Materials and methods

### Patient population

**FIGURE 1:**
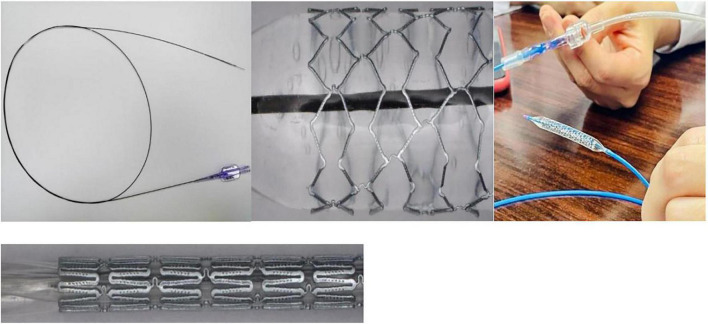
IBS^®^ Angel™ Stent system.

From July 2021 to February 2022, we prospectively evaluated the efficacy and safety of stenting for branch pulmonary artery stenosis using the biodegradable nitride iron stent IBS^®^ Angel™ (Biotyx, Shenzhen, China) (Figure 1) and right ventricular systolic and diastolic function. A total of eleven cases were included. This was a pre- and post-intervention, prospective cohort study. The clinical trial was approved by the Clinical Research Ethic Committee of Guangdong Provincial People’s Hospital (ethical approval number: GDREC 2019-99).

The inclusion criteria were as follows: (1) patients must between 1 and 14 years old; (2) patients needed to have congenital or secondary pulmonary artery stenosis (including the pulmonary artery trunk, left pulmonary artery and right pulmonary artery), and meet one of the following criteria: (A) the pressure gradient across the stenosis was ≥ 20 mmHg as measured by a catheter; (B) the degree of pulmonary artery stenosis was ≥ 50% (as determined calculated by the following formula: diameter of adjacent normal segments - residual lumen diameter of the stenosis segment)/(diameter of adjacent normal segments × 100%); (C) the ratio of right ventricular systolic pressure to aortic systolic pressure was ≥ 50%; (3) patients and their families were compliant with the requirements of the study, and were willing to participate voluntarily, sign the informed consent form, complete the 2-year follow-up; and (4) patients had a life expectancy of > 2 years after successful stent implantation.

The exclusion criteria were as follows: (1) patients who had a history of disease related to iron overload or iron disorder, such as hereditary hemochromatosis; (2) patients with cardiopulmonary function that could not tolerate surgery, such as those with severe heart failure (NYHA Grade III and above) that could not be controlled by active medical treatment; (3) patients with known allergies to contrast agent, iron and its degradation products; (4) patients with hemorrhagic disorders; (5) patients with contraindications to antiplatelet agents and anticoagulant therapy; (6) patients with thrombosis at the vascular wall of the target lesion or the distal or proximal location; (7) patients with confirmed severe renal or hepatic insufficiency who were unsuitable for the index procedure according to the investigator’s judgment; (8) previous stent implantation had been performed to treat the target lesion; (9) patients with severe stenosis or excessive tortuosity in the targeted vessels, or anatomical abnormalities, thus making it difficult for the device to reach the target lesion; (10) patients with other conditions that are not suitable for stent delivery or balloon expansion; (11) patients who have already participated in another drug or medical device clinical trial that have not yet completed or withdrawn within 3 months before the screening period of the current trial; and (12) patients who were not suitable for participating in the trial as per the investigator’s judgment.

### Method of implantation

The biodegradable nitride iron stent IBS^®^ Angel™ (Biotyx, Shenzhen, China) have different sizes. The diameters of the stents range from 2.25 to 10 mm. The lengths of the stents range from 8 to 38 mm. The sizes of the delivery long sheath range from 4 to 9F. The radial support of the stent ranges from 90 to 140 kpa. All patients underwent right heart catheterization under general anesthesia. A TERUMO 6F (Japan) femoral sheath was used to puncture the right or left femoral vein. The intravenous dose was 100 U/kg of heparan. The right ventricle, pulmonary artery trunk, and the distal branch of the pulmonary artery pressure were measured with a TERUMO 6F-MPA2-100 (Japan) catheter along the right femoral vein, the right atrium, the right ventricle, the pulmonary artery trunk, the branch pulmonary artery. Then, we calculated the pressure gradient across the stenosis. Right ventricle or main pulmonary artery (mPA) angiography was performed in anteroposterior, lateral and cephalic positions at 15°–20° to assess the morphology of stenosis in the branch pulmonary artery; for this, we used a TERUMO 5F-pigtail (Japan) catheter. Care was taken to note the position of the branching vessels and their relationship to the stenosis. Following pulmonary artery angiography, different size stents were mounted on balloons and directed to straddle the lesions with an 6F or 8F long sheath guide (Cook Inc) over a 0.035-inch extra-stiff exchange interventional wire (Amplatz type, Cook Inc). Following placement, we attempted to dilate both the proximal and distal pulmonary vessels. After catheterization, low molecular weight heparin sodium was injected subcutaneously twice at 100 U/kg^–1^ after 4 and 16 h after intervention. Acetylsalicylic acid (3 to 5 mg/kg^–1^/d^–1^) was prescribed for 6 months, and clopidogrel (1 mg/kg^–1^/d^–1^) was prescribed for 1 month.

### Follow-up protocol

Prior to stent implantation, patients underwent routine transthoracic echocardiographic (TTE), chest radiography and computed tomography (CT) with a 256-slice scanner equipped with a multiple detector [multiple-detector computed tomography (MDCT); Brilliance iCT; Philips Healthcare, Cleveland, OH, United States]. Blood was also drawn so that we could determine the levels of iron metabolism indicators. TTE examination was carried out preoperatively using IE33, IE Epic-7C (Philips, Andover, MA, United States) and Vivid E 9 (GE Healthcare, Horten, Norway) ultrasound systems with a transducer of 5–8 MHz. The follow-up period was 2 years. Following implantation, we determined pulmonary artery perfusion (the diameters of the stents and the pressure gradients across the stents) and right ventricular function parameters [tricuspid annular plane systolic excursion (TAPSE), pulsed Doppler S wave (S’), fractional area change (FAC), the ration of E/A, E′/A′ and E/E′] with TTE on day 1, month 1,3,6,12,24. The morphology and degradation of the stent were evaluated by MDCT one year after implantation. When suspected stent stenosis was confirmed by echocardiography within 1,3,6 months after implantation, MDCT examination was performed, and angiography was necessary. Iron metabolism, liver and kidney function and coagulation indexes were examined half a year after operation. The Pre- and post-operative TTE images and MDCT images were reviewed by two independent experienced pediatric cardiologists to determine the location and morphology of the stenosis in the branch pulmonary artery.

The primary end point was composite, combining major adverse cardiovascular events that included cardiovascular mortality, in-stent thrombosis, vascular embolism, stent displacement, and heart failure. Secondary endpoints were branch pulmonary artery diameter evolution, complications related to the procedure, intervention rate, and all-cause mortality.

### Statistical analyses

Continuous variables are expressed as mean standard ± deviation or median with range. Categorical variables are presented as numbers and proportions, and between-group differences were analyzed using the Student’s *t*-test or the Mann-Whitney U test as appropriate. All analyses were performed using SPSS Statistics 23 (IBM, Chicago, IL, United States) and a two-sided *P* < 0.05 was considered as statistical significance.

## Results

### Clinical characteristics

**FIGURE 2:**
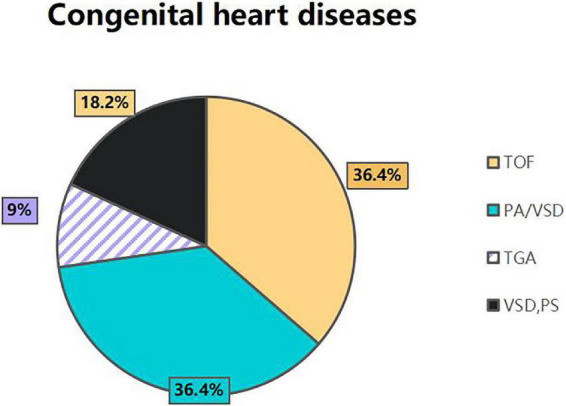
The distributions of congenital heart diseases in 11 patients.

A total of 11 children with PS were included in the current analysis. There were two female patients and nine male patients. Ages ranged from 36 to 86 months (mean age: 59.7 ± 16.05 months). Weight ranged from 14 to 20 kg (mean weight: 16.8 ± 1.94 kg). All of the children with PS had congenital heart diseases; the most commonly detected abnormalities were tetralogy of Fallot (TOF) and pulmonary atresia with ventricular septal defect (PA/VSD), both of which were found in four (36.4%) patients, followed by VSD combined PS in two (18.2%), and complete transposition of the great artery (TGA) in one (9%) patient (Figure 2). All patients had undergone surgery or balloon angioplasty more than one time. However, the effects were unsatisfactory and it had proved difficult to resolve the stenosis in the distal branch of the pulmonary artery. These patients were enrolled for the current study after written and informed consent had been obtained from the parents. Following surgery for congenital heart disease, the remaining stenosis in the branch pulmonary artery was mainly in the left pulmonary artery in eight cases (8/11), the right pulmonary artery in two, and the left and right pulmonary artery in one case (Tables 1, 2).

**TABLE 1:** Clinical characteristics.

Case	Sex (F/M)	Age (m)	Weight (kg)	Diagnosis	Operation	Operation frequency
Case 1	M	55	19	PA/VSD(I)	Pulmonary artery autologous pericardium repair, VSD repair, RVOT dredging, PDA ligation, ASD repair	1
Case 2	M	76	20	PA/VSD(III)	AP shunt, Melbourne shunt, RVOT reconstruction (pericardial duct), pulmonary valve reconstruction (artificial single valve), VSD repair, atrial septostomy, pulmonary stenosis correction	3
Case 3	M	86	17.5	VSD, PS, LPS	Correction of pulmonary artery stenosis, left pulmonary artery opening formation, VSD repair, single pulmonary valve formation	2
Case 4	M	45	17	TOF	Correction of tetralogy of Fallot, ASD repair, PDA ligation, percutaneous balloon dilation of the left pulmonary artery	2
Case 5	M	36	14.5	TGA/IVS	Switch operation	1
Case 6	F	51	16	PA/VSD(I)	pulmonary valve reconstruction (artificial single valve), VSD repair	1
Case 7	M	46	14	PA/VSD(I)	RVOT reconstruction (valved homograft conduit), percutaneous balloon dilation of the left pulmonary artery, valve replacement of pulmonary artery	3
Case 8	M	71	17	TOF	Correction of tetralogy of Fallot	1
Case 9	F	74	18.5	VSD, LPS, RPS	left and right pulmonary artery formation, VSD repair, PDA ligation, ASD repair, percutaneous balloon dilation of the left pulmonary artery, stent implantation of right pulmonary artery	2
Case 10	M	48	14.5	TOF, PAPVC	Correction of tetralogy of Fallot and PAPVC	1
Case 11	M	69	17	TOF	Correction of tetralogy of Fallot	1

F, female; M, male; m, month; PA/VSD (I/III), pulmonary atresia with ventricular septal defect (I/III type); TOF, tetralogy of Fallot; RPS/LPS, right or left pulmonary artery stenosis; PAPVC, partial anomalous pulmonary venous connection; ASD, atrial septal defect; PDA, patent ductus arteriosus; RVOT, right ventricular outflow tract; TGA/IVS, complete transposition of the great artery/integrity of ventricular septum.

**TABLE 2:** 256-slice scanner multiple-detector computed tomography (MDCT) parameters and morphological manifestations.

Case	Lesion vessels	A1 (mm)	A2 (mm)	A3 (mm)	L (mm)	Lesion location
Case 1	LPA	3.02	6.575	7.254	10.417	middle
Case 2	LPA	3.699	10.058	6.381	9.653	middle
Case 3	LPA	1.819	1.819	6.773	10.973	opening
Case 4	LPA	0.86	0.86	3.77	13.827	opening
Case 5	RPA	3.01	5.52	6.42	21.92	middle
Case 6	LPA	2.887	2.887	6.854	13.79	opening
Case 7	RPA	2.85	4.28	8.09	23.28	middle
Case 8	LPA	3.667	3.667	10.222	11.866	opening
Case 9	LPA	3.76	6.16	5.07	17.46	middle
Case 10	LPA	4.714	5.053	8.082	9.16	middle
	RPA	2.893	3.012	9.202	15.77	middle
Case 11	LPA	2.28	2.28	10.31	23.73	opening

A1, Narrowest diameter; A2, Proximal diameter; A3, Distal diameter; L, The length of the narrow.

Blood samples were taken from each child to determine the levels of indicators for iron metabolism. The mean serum level of iron was 13.6 ± 5.5 μmol/L; the mean serum levels of transferrin and ferritin were 2.5 ± 0.35 g/L and 66.2 ± 58.9 ng/ml, respectively. Preoperative indices of iron metabolism indices were normal. Since the other patients did not reach the 6-month follow-up period, indices of iron metabolism were measured again in only 3 patients at 6-month follow-up; no significant abnormalities were found.

### Morphological analysis of lesion vessels prior to intervention

**FIGURE 3:**
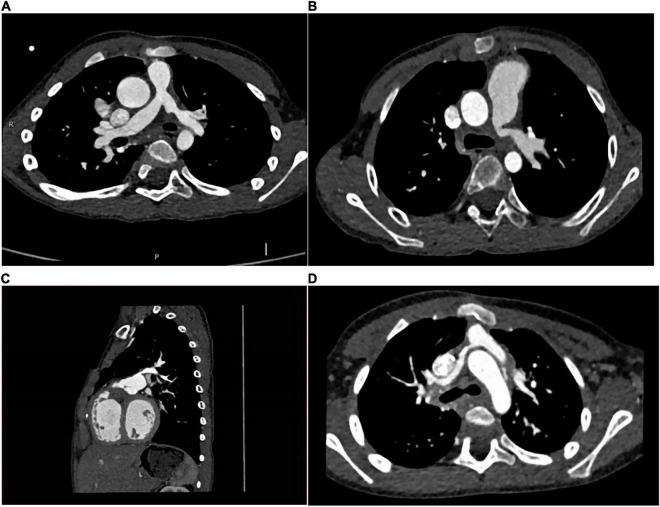
MDCT images of cases. **(A)**: case 1 had PA/VSD, LPS; **(B)**: case 8 had TOF, LPS; **(C)**: case 3 had VSD, LPS; **(D)**: case 5 had TGA, RPS.

**FIGURE 4:**
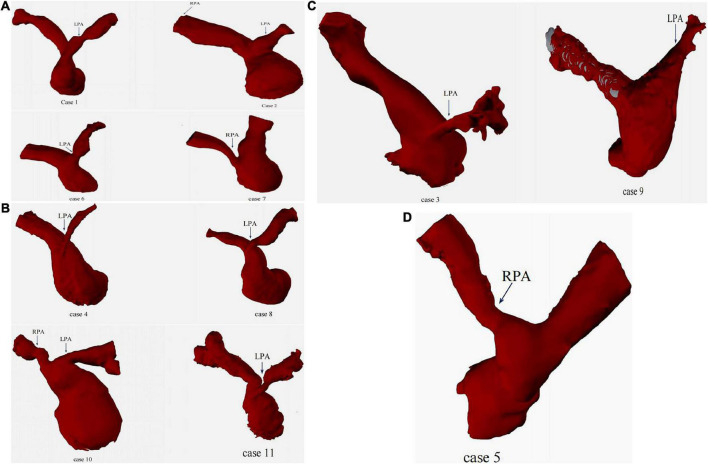
3D images of branch pulmonary artery. **(A)**: PA/VSD group; **(B)**: TOF group; **(C)**: VSD/PS group; **(D)**: TGA group.

Transthoracic echocardiographic (TTE) and MDCT was carried out preoperatively in 11 cases. The parameters measured in MDCT included: A1 (narrowest diameter), A2 (proximal diameter), A3 (distal diameter) and L (the length of the lesion). The narrowest diameters of the lesion vessels ranged from 0.86 to 4.714 mm (mean diameter: 2.95 ± 0.998 mm), the proximal diameters ranged from 0.86 to 10.06 mm (mean diameter: 4.35 ± 2.52 mm), the distal diameters ranged from 3.77 to 10.31 mm (mean diameter: 7.37 ± 1.95mm), and the length of the lesion ranged from 9.16 to 23.73 mm (mean length: 15.15 ± 5.32 mm) (Table 2). According to MDCT images (Figure 3), the morphological manifestations of stenosis in the branching pulmonary artery were as follows: cases 3, 4, 6, 8, and 11 presented with opening stenosis while the remaining six cases mainly presented with middle branch stenosis. We also established three-dimensional (3D) images of the branch pulmonary artery based on MDCT images to intuitively understand the spatial structure of the branch pulmonary artery. The 3D structural profiles of the 11 cases of branch pulmonary arteries are shown in Figure 4. These images were divided into four groups according to the type of congenital heart disease. In the PA/VSD group, three cases presented with stenosis in the left branch of the pulmonary artery and one presented with stenosis in the right branch of the pulmonary artery. All cases in the TOF and VSD/PS group presented with stenosis in the left branch of the pulmonary artery. In the TGA group, one case presented with stenosis in the right branch of the pulmonary artery.

### Right heart catheterization and stent implantation

**FIGURE 5:**
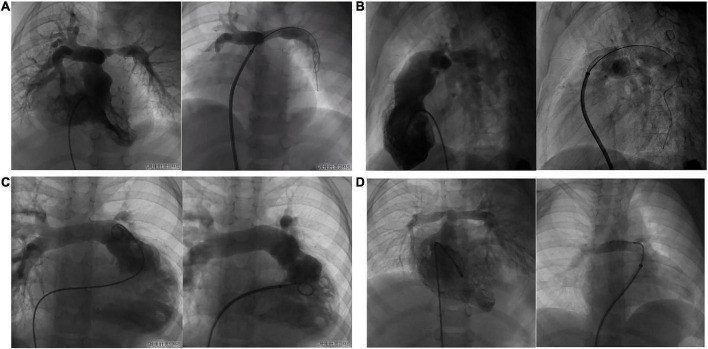
The DSA images of before and after pulmonary artery stenting. **(A)**: case 1 had PA/VSD, LPS and implanted IBS; **(B)**: case 8 had TOF, LPS and implanted IBS; **(C)**: case 3 had VSD, LPS and implanted IBS; **(D)**: case 5 had TGA, RPS and implanted IBS.

Eleven cases underwent right heart catheterization under general anesthesia. The measured parameters included: A1 (narrowest diameter), L (length of the lesion vessels) and A2 (distal normal diameter of the lesion vessels). The hemodynamic parameters included: preoperative/postoperative right ventricle (RV) pressure, main pulmonary artery (mPA) pressure, and pressure gradient (Table 3). Nine patients had stenting in the left pulmonary artery, and three had stenting in the right pulmonary artery. The DSA images before and after pulmonary artery stenting are shown in Figure 5. The diameters of the stents were 10 mm in three patients, 8 mm in six patients, 6 mm in two patients, and 5 mm in one patient. The lengths of the stents were 23 mm in nine patients, 28 mm in two patients, and 18 mm in one patient. The sizes of the delivery sheath used ranged from 6 to 8F and the balloon pressures ranged from 12 to 16 atm.

**TABLE 3:** Heart catheterization evaluation and hemodynamic parameters.

Case	Lesion vessels	A1 (mm)	A2 (mm)	L (mm)	Preoperative RV pressure (mmHg)	Preoperative mPA pressure (mmHg)	Preoperative left/right distal PA pressure (mmHg)	Pressure gradient (mmHg)	Stent (mm)	Postoperative RV pressure (mmHg)	Postoperat-ive mPA pressure (mmHg)	Pressure gradient (mmHg)	Balloon pressure
Case 1	LPA	4.24	8.4	18.14	85/3(30)	85/3(30)	21/8(12)	64	8*23	58/7(24)	58/7(24)	33	12atm
Case 2	LPA	4.8	8.9	23.2	76/4(28)	63/10(28)	25/13(17)	38	8*23	64/9(27)	55/10(25)	24	12atm
Case 3	LPA	1.9	6.2	24.1	55/2(20)	50/12(24)	—	—	6*23	29/0(9)	—	—	12atm
Case 4	LPA	0.9	3.5	10.8	50/1(17)	46/4(18)	22/13(16)	24	5*28	35/0(11)	34/7(16)	9	14atm
Case 5	RPA	3.2	10.12	16	80/0(27)	76/24(41)	19/9(12)	57	8*18	48/-1(15)	48/10(22)	23	16atm
Case 6	LPA	3.3	6.8	16.7	63/0(21)	38/11(20)	14/4(7)	24	8*23	—	50/1(17)	14	15atm
Case 7	RPA	2.39	8.0	15	53/0(17)	33/10(17)	12/10(10)	21	8*28	30/0(10)	30/3(12)	8	14atm
Case 8	LPA	3.2	9.0	16.19	90/0(30)	70/25(45)	36/12(20)	34	8*23	53/0(17)	53/12(25)	17	15atm
Case 9	LPA	3.1	4.9	12	90/1(33)	90/14(39)	28/13(18)	62	6*23	56/6(29)	55/9(24)	16	16atm
Case 10	LPA RPA	4.7 4.6	10 9.2	20 16	100/0(33)	87/10(32)	27/15(19) 13/4(7)	60 74	10*23 10*23	34/3(13)	33/6(15)	14 11	14atm 14atm
Case 11	LPA	4.06	11	16.1	65/3(24)	—	25/10(15)	40	10*23	48/1(18)	48/10(22)	7	14atm

A1, Narrowest diameter; A2, Distal diameter; L, The length of the narrow; RV, right ventricle; mPA, main pulmonary artery pressure. *Diameter and length.

With regards to the primary end point, stenting was successful in 11 patients. There were no major adverse cardiovascular events related to the device or to the procedure. With regards to secondary end points, as the narrow pulmonary artery was expanded, the blood perfusion in the branch pulmonary artery improved immediately. The narrowest mean diameter of the lesion vessels was 3.37 ± 1.2 mm before stenting; these were dilated to 7.92 ± 1.62 mm after stenting. There was a significant difference in the immediate postoperative stenting effect in terms of the diameter of the lesion vessels and the pressure gradient across the stenosis (*p* < 0.05). Preoperative mean pressure gradient across the stenosis was 44.5 ± 22.1 mmHg; this decreased to 14.7 ± 8.9 mmHg after stenting. Preoperative mean RV systolic pressure and mPA systolic pressure were 73.36 ± 17.10 mmHg and 63.80 ± 21.04 mmHg, respectively; these decreased to 45.50 ± 12.62 mmHg and 46.40 ± 10.26 mmHg, respectively, after intervention and were both statistically significant (*p* < 0.01 and *p* = 0.03, respectively).

### Transthoracic echocardiographic evaluation of stenting efficiency

Eleven cases underwent TTE examination preoperatively and at follow-up intervals. The narrowest mean diameter of the lesion vessels was 4.58 ± 1.92 mm before stenting and the mean pressures gradient across the stenosis was 48.5 ± 26.9 mmHg. After 1 day, 1 month, and 3 months, we evaluated the diameters of the lesion vessels and the pressure gradients across the stents (Tables 4, 5). The diameters of the lesion vessels were 7.02 ± 1.6, 7.57 ± 4.1, and 9.07 ± 9.2 mm, respectively, and the pressure gradients across the stenting were 25.33 ± 16.9, 31.83 ± 17.97, and 24.83 ± 13.26 mmHg, respectively. The diameters of the vessels were significantly dilated when compared with that before stenting (*p* = 0.011, 0.008, and 0.011, respectively) and the pressure gradients across the target vessels were significantly reduced after one day and three months when compared with that before stenting (*p* = 0.021 and 0.037). Furthermore, there was no significant difference in the vascular diameters and pressure differences between groups when tested 1 day, 1 month and three months after stenting (*p* = 0.642 and 0.445), thus indicating that no significant restenosis was detected during follow-up that required re-intervention. None of the patients experienced in-stent thrombosis, vascular embolism, stent displacement, or heart failure.

**TABLE 4:** Echocardiographic vessels parameters in 11 cases.

Case	Lesion vessels	A0 (mm)	P0 (mmHg)	A1 (mm)	P1 (mmHg)	A2 (mm)	P2 (mmHg)	A3 (mm)	P3 (mmHg)	A4 (mm)	P4 (mmHg)
Case 1	LPA	4.7	82	7.7	61	6.3	64	7.9	38	8	29
Case 2	LPA	4.7	61	7.8	20	7	45	6.1	40	5.4	45
Case 3	LPA	1.6	-	4.7	41	4.7	41	4.8	18	4.5	21
Case 4	LPA	2.9	16	4.1	9	4.2	9	4.1	11	–	–
Case 5	RPA	4.8	61	7.4	21	6.8	40	6.5	31	–	–
Case 6	LPA	4.7	59	7.3	39	7.8	44	7.5	38	–	–
Case 7	RPA	3.7	46	7.8	13	6.2	37	6.5	22	–	–
Case 8	LPA	4	36	7.7	21	6.5	3	7.4	3	–	–
Case 9	LPA	2.7	90	4.6	8	5.2	15	4.3	9	–	–
Case 10	LPA RPA	9 5.7	21 67	8.4 8.8	7 8	8.1 8.1	20 20	7.4 8.3	24 38	– –	– –
Case 11	LPA	6.5	43	9	39	9	41	9	48	–	–

A0, Preoperative narrowest diameter of target vessel stenosis; P0, Preoperative pressure gradient across stenosis; A1, Postoperative one day diameter of target vessel stenosis; P1, Postoperative one day pressure gradient across stenosis; A2, Postoperative one month diameter of target vessel stenosis; P2, Postoperative one month pressure gradient across stenosis; A3, Postoperative three month diameter of target vessel stenosis; P3, Postoperative three month pressure gradient across stenosis; A4, Postoperative six month diameter of target vessel stenosis; P4, Postoperative six month pressure gradient across stenosis.

**TABLE 5:** Transthoracic echocardiographic (TTE) evaluation the effective stenting.

			*P*-value
Preoperative	A0 (mm)	4.58 ± 1.92	–
	P0 (mmHg)	48.5 ± 26.9	–
Postoperative one day	A1 (mm)	7.02 ± 1.6	0.011*
	P1 (mmHg)	25.33 ± 16.9	0.021*
Postoperative one month	A2 (mm)	7.57 ± 4.1	0.008*
	P2 (mmHg)	31.83 ± 17.97	0.083
Postoperative three month	A3 (mm)	9.07 ± 9.2	0.011*
	P3 (mmHg)	24.83 ± 13.26	0.037*

A0, Preoperative narrowest average diameters of the lesion vessels; P0, Preoperative average pressures gradient across stenosis; A1, Postoperative one day average diameters of the lesion vessels; P1, Postoperative one day average pressures gradient across stenosis; A2, Postoperative one month average diameters of the lesion vessels; P2, Postoperative one month average pressures gradient across stenosis; A2, Postoperative three month average diameters of the lesion vessels; P2, Postoperative three month average pressures gradient across stenosis; *: P < 0.05.

### Transthoracic echocardiographic evaluation of right heart function

**FIGURE 6:**
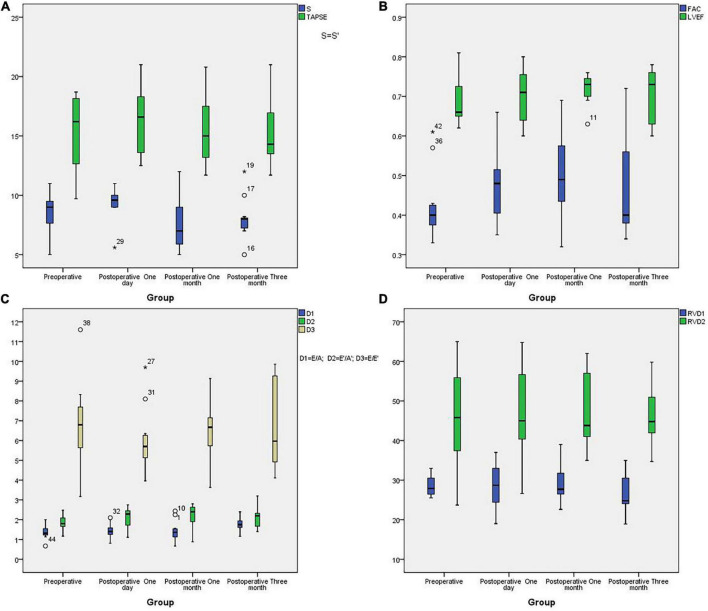
The boxplots of echocardiographic parameters. **(A)**: The boxplots of S’ and TAPSE in different groups; **(B)**: The boxplots of LVEF and FAC in different groups; **(C)**: The boxplots of E/A, E′/A′ and E/E′ in different groups; **(D)**: The boxplots of RVDA and RVD2 in different groups.

All patients underwent TTE examination to evaluate right heart function before stenting and during the three months follow-up interval after stenting. There are not many reference indicators for children. According to “Echocardiographic assessment of the right heart in adults,” a practical guideline from the British Society of Echocardiography in 2020 (12), we chose some parameters to reflect right ventricular diastolic function, including Doppler interrogation of tricuspid inflow (the E/A ratio) and Tissue Doppler at the lateral tricuspid annulus (E′/A′ and E/E′ratio). We also chose some parameters to reflect right ventricular systolic function, including tricuspid annular plane systolic excursion (TAPSE), pulsed Doppler S wave (S’) and fractional area change (FAC). An RV FAC ≥ 30% in males or ≥ 35% in females is considered normal. A TAPSE < 1.7 cm is highly suggestive of RV systolic dysfunction. An S’wave velocity ≥9 cm/s indicates a normal RV long axis systolic function. The normal range for the TV E/A ratio is 0.8–2.1 while a TV E/A < 0.8 may indicate impaired RV relaxation. A TV E/A > 2.1 may indicate restrictive RV filling while an E′/A′ < 1 is abnormal and an E/E′ > 6 suggests elevated RA pressure. We also measured RVD1; this represents the mid RV diameter measured at the level of the RV papillary muscles. An RVD1 ≤ 42 mm in males or ≤ 35 mm in females, is considered normal. We also determined the RVD2:RV length, taken as the plane of the tricuspid annulus to the RV apex. An RVD2 ≤ 87 mm in males or ≤ 80 mm in females is considered normal. Echocardiographic parameters for the 11 patients are given in Table 6 while boxplots are shown in Figure 6. Right ventricular systolic function parameters prior to stenting were as follows: S’ wave velocity ranged from 5 to 11 cm/s (mean: 8.44 ± 1.78 cm/s), TAPSE ranged from 9.7 to 18.7 mm (mean: 15.23 ± 3.45mm), and FAC ranged from 33 to 61% (mean: 42.2% ± 8.82%). Right ventricular diastolic function parameters prior to stenting were as follows: E/A ratio ranged from 0.67 to 2.00 (mean: 1.37 ± 0.35), E′/A′ ratio ranged from 1.17 to 2.48 (mean: 1.84 ± 0.41), E/E′ ratio ranged from 3.17 to 11.60 (mean: 6.66 ± 2.29). Parameters of right atrial and right ventricle cardiac cavity size were as follows: right atrial transverse diameter ranged from 23.5 to 43.00 mm (mean: 30 ± 5.95 mm), RVD1 ranged from 25.5 to 33.0 mm (mean: 28.65 ± 2.61 mm), and RVD2 ranged from 23.7 to 65.00 mm (mean: 46.1 ± 12.6 mm). RVD1/RVD2 ranged from 0.46 to 1.11 (mean: 0.67 ± 0.21) and LVEF ranged from 64 to 81% (mean: 68.9% ± 5.96%). All of the patients had normal left ventricular systolic function. It was difficult to satisfy all of the indicators children’s right heart function because there are no gold standards. Compared with normal values, there were no statistical with regards to S’, TAPSE, or E/E′ for preoperative tests or tests carried out on day 1, month 1 and month 3 after implantation. However, significant differences were identified for FAC, E/A and E′/A′ (*p* < 0.01) (Table 7). These results showed that the 11 cases had normal right ventricular systolic and diastolic function before and after stenting. We also found that there was no statistical difference in any of the ultrasound parameters when compared before stenting and day 1 post-stenting. One month post-stenting, FAC and E′/A′ were statistically different from those before stenting (*p* = 0.041 and *p* = 0.035). Three months post-stenting, only E/A showed a statistical difference (*P* = 0.015) (Table 6).

**TABLE 6:** Echocardiographic parameters about right and left ventricular function.

	Preoperative	Postoperative one day	*P*-value	Postoperative one month	*P*-value	Postoperative three month	*P*-value
S’ cm/s	5–11 (8.44 ± 1.78)	5.6–11 (9.29 ± 1.38)	0.142	5–12 (7.48 ± 2.31)	0.262	5–12 (8.02 ± 1.78)	0.277
TAPSE mm	9.7–18.7 (15.23 ± 3.45)	12.5–21 (16.45 ± 2.87)	0.49	11.7–20.8 (15.55 ± 3.02)	0.921	11.7–21 (15.25 ± 2.97)	0.948
FAC	33%–61% (42.2% ± 8.82%)	35%–66% (47.6% ± 9.44%)	0.167	32%–69% (50.6% ± 11.56%)	0.041*	34%–72% (47.6% ± 13.5%)	0.39
E/A	0.67–2 (1.37 ± 0.35)	0.81–2.1 (1.43 ± 0.39)	0.576	0.67–2.44 (1.43 ± 0.52)	0.974	1.16–2.4 (1.77 ± 0.35)	0.015*
E′/A′	1.17–2.48 (1.84 ± 0.41)	1.11–2.75 (2.11 ± 0.53)	0.25	0.88–2.8 (2.24 ± 0.57)	0.035*	1.4–3.2 (2.07 ± 0.52)	0.411
E/E′	3.17–11.6 (6.66 ± 2.29)	3.96–9.7 (5.96 ± 1.68)	0.309	3.63–9.14 (6.44 ± 1.54)	0.818	4.11–9.86 (6.76 ± 2.22)	0.974
RA mm	23.5–43 (30 ± 5.95)	20–42 (28.6 ± 6.89)	0.357	21–42 (29.47 ± 6.98)	0.669	24–34 (28.2 ± 3.19)	0.645
RVD1 mm	25.5–33 (28.65 ± 2.61)	19–37 (28.5 ± 5.76)	0.743	22.6–39 (29.12 ± 4.44)	0.922	18.9–35 (27.04 ± 4.94)	0.25
RVD2 mm	23.7–65 (46.1 ± 12.6)	26.6–64.8 (46.8 ± 12.15)	0.895	35–62 (47.34 ± 9.55)	0.742	34.7–59.8 (46.02 ± 7.39)	0.922
RVD1/RVD2	0.46–1.11 (0.67 ± 0.21)	0.37–0.98 (0.63 ± 0.15)	0.921	0.44–0.83 (0.63 ± 0.11)	0.947	0.45–0.74 (0.59 ± 0.09)	0.535
LVEF	64%–81% (68.9% ± 5.96%)	60%–80% (70.3% ± 7.29%)	0.693	63%–76% (71.8% ± 3.71%)	0.166	60%–78% (70.1% ± 6.89%)	0.818

*P < 0.05.

**TABLE 7:** Echocardiographic parameters about right ventricular function.

	Normal value	Preoperative P	Postoperative one day P	Postoperative one month P	Postoperative three month P
S’ cm/s	≥ 9	0.319	0.5	0.054	0.097
TAPSE mm	≥ 17	0.119	0.537	0.143	0.08
FAC	≥ 30%	0.001*	<0.01*	<0.01*	0.001*
E/A	0.8–2.1	<0.01*	<0.01*	0.003*	<0.01*
E′/A′	≥ 1	<0.01*	<0.01*	<0.01*	<0.01*
E/E′	≤ 6	0.359	0.943	0.368	0.285

*P < 0.05.

**FIGURE 7:**
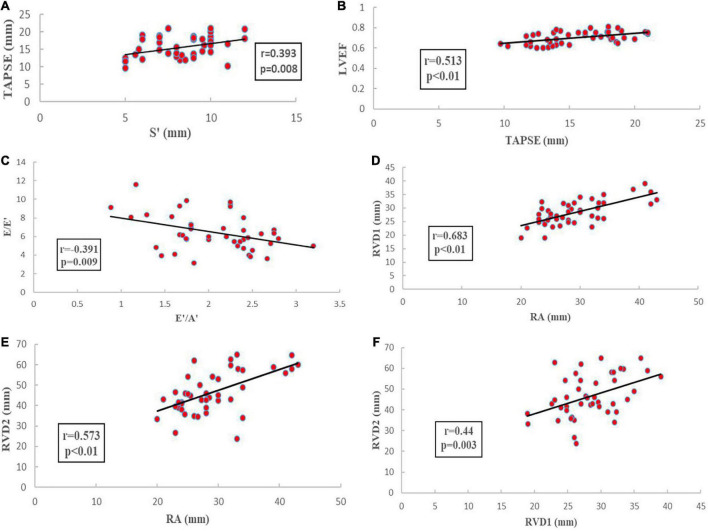
Echocardiographic parameters correlated analysis. **(A)**: the correlation analysis of TAPSE and S’; **(B)**: the correlation analysis of TAPSE and LVEF; **(C)**: the correlation analysis of E/E′ and E′/A′; **(D)**: the correlation analysis of RA and RVD1; **(E)**: the correlation analysis of RA and RVD2; **(F)**: the correlation analysis of RVD1 and RVD2.

Based on correlation analysis of echocardiographic parameters, TAPSE correlated significantly with S’ (*r* = 0.393; *p* = 0.008) and LVEF (*r* = 0.513; *p* < 0.01), E/E′ correlated significantly with E′/A′ (*r* = 0.391; *p* = 0.009), RA volume correlated significantly with RVD1 and RVD2 volume (*r* = 0.44; *p* = 0.003 and *r* = 0.573, *p* < 0.01, respectively), and RVD1 volume correlated significantly with RVD2 volume (*r* = 0.44, *p* = 0.003) (Figure 7).

Table 8 shows tricuspid and pulmonary valve regurgitation. Before stenting, no tricuspid valve regurgitation was in three cases while mild tricuspid valve regurgitation was observed in eight cases. Three months after stenting, limited tricuspid valve regurgitation was observed in two cases, mild tricuspid valve regurgitation in seven cases, and moderate tricuspid valve regurgitation were in two cases. Due to the complex nature of congenital heart disease, almost all patients received pulmonary valve reconstruction including valve replacement. Therefore, 11 cases had pulmonary valve regurgitation of varying degrees. Prior to stenting, no pulmonary valve regurgitation was observed in one case, mild pulmonary valve regurgitation in one case, mild-moderate pulmonary valve regurgitation in two cases, moderate pulmonary valve regurgitation in four cases, moderate-severe pulmonary valve regurgitation in one case, and severe pulmonary valve regurgitation in two cases. Three months after stenting, no pulmonary valve regurgitation was observed in one case, limited pulmonary valve regurgitation in one case, mild pulmonary valve regurgitation in one case, moderate pulmonary valve regurgitation in three cases, moderate-severe pulmonary valve regurgitation in four cases, and severe pulmonary valve regurgitation in one case. There were no significant differences in tricuspid and pulmonary valve regurgitation compared to before stenting.

**TABLE 8:** The conditions of tricuspid and pulmonary valve regurgitation.

Case	Preoperative TR	Preoperative PR	Postoperative one day TR	Postoperative one day PR	Postoperative one month TR	Postoperative one month PR	Postoperative three month TR	Postoperative three month PR
1	Mild	Moderate	Mild	Moderate	Mild	Moderate-severe	Mild	Moderate-severe
2	Mild	Moderate	Mild	Moderate	Mild	Moderate	Moderate	Moderate
3	Mild	Moderate-severe	No	Moderate-severe	Mild	Moderate-severe	Mild	Moderate-severe
4	Mild	Moderate	Mild	Moderate	Moderate	Moderate	Moderate	Moderate
5	No	No	No	No	No	No	Limited	No
6	Mild	Moderate	Mild	Moderate	Mild	Moderate	Mild	Mild
7	No	Mild-moderate	Mild	Moderate	Mild	Moderate	Mild	Moderate
8	Mild	Mild-moderate	Mild	Mild-moderate	Mild	Mild-moderate	Mild	Mild-moderate
9	No	Mild	No	No	Mild	Limited	Limited	Limited
10	Mild	Severe	Mild	Severe	Mild	Severe	Mild	Moderate-severe
11	Mild	Severe	Mild	Severe	Mild	Severe	Mild	Severe

TR, tricuspid valve regurgitation; PR, pulmonary valve regurgitation.

## Discussion

Primary or secondary branch pulmonary artery stenosis complicates the management of congenital heart diseases. Tetralogy of Fallot and pulmonary atresia with ventricular septal defect are the most common congenital heart diseases involved. This is consistent with our own clinical trials results. Over the past several decades, technological advances and innovative approaches have expanded our range of therapeutic options for percutaneous catheterization for branch pulmonary artery stenosis (13–15). However, the major issue associated with stenting in young children is the need for stent re-dilatation to adapt to the child’s vascular growth (16, 17). Another issue is intrastent proliferation, which in some cases leads to severe obstruction (18, 19). To solve these problems, significant advancements have been made with metallic bioresorbable stents because of their advantages over permanent stents; for example, the prevention of chronic injury and inflammatory reactions, and the reduction of late in-stent thrombosis (20). A biodegradable stent is the perfect choice for infants or children with primary or secondary branch pulmonary artery stenosis.

The biodegradable nitride iron stent (IBS^®^ Angel™) has shown excellent mechanical performance and biocompatibility (21). The IBS design incorporates a surface modification technique in that plasma nitriding was applied to the thin-wall pure iron stent, making this one of the thinnest bioresorbable stents in the world. Furthermore, it has a comparable low crossing-profile, 5 French guiding catheter compatibility, mechanical strength, a specification range, endothelialization capability, safety, and effectiveness for state-of-the-art permanent drug-eluting stents both *in vitro* and in animal models (22, 23). However, clinical trials have yet to demonstrate the efficacy and safety of IBS for branch pulmonary artery stenosis in children. Our clinical trials first demonstrated the efficacy and safety of IBS for branch pulmonary artery stenosis in children in initial 3-month. The mean diameter of the narrowed branch pulmonary arteries encountered in our patients was 3.37 ± 1.2 mm; this was significantly dilated to 7.92 ± 1.62 mm after stenting. RV and mPA systolic pressures were significantly reduced after stent implantation. Furthermore, over a three-month follow-up period, transthoracic echocardiographic indicated that the intra-stent blood flow was unobstructed without stent thrombosis, displacement, or restenosis. There were no serious adverse events related to the device or to the procedure. The technique was feasible and permitted the use of a small size 6-8 French delivery sheath, which does not damage the blood vessels of children. Short-term data have demonstrated excellent results for bare metal stents or drug eluting stents when implanted in branch pulmonary arteries; however, reintervention is the primary failure method with the extended follow-up. Recent observational studies demonstrated a high rate of reintervention of 60% at 10 years (24) and 65% at 5 years for permanent stents (25). Compared with permanent stents, bioresorbable stents are the direction of future research. Currently, magnesium-based (Mg) stent devices remain at the forefront of bioresorbable stent material development and use. In 2005, the first preclinical trial implanted Absorbable Metal Stents (AMS) (Biotronik, Berlin, Germany) into the infra-popliteal arteries of 20 patients with critical limb ischemia (26). The results showed that a total of 13 patients had normal blood flow, 4 had partial stenosis, and 2 had complete occlusion at 3 month follow-up, as determined by duplex doppler ultrasound. Subsequently, new Mg-based stent devices, such as DREAMS, DREAMS 2G, and Magmaris were used for the treatment of coronary artery disease. These clinical trials have demonstrated high procedural success and safety rates accompanied by low rates of stent thrombosis and malposition (27–29). However, Mg-based bioresorbable stents have some drawbacks, including rapid degradation time and scaffold disappearance; furthermore, Mg materials have lower radial strength, thus leading to higher vessel recoil, late lumen loss, and larger struts (30). These challenges have intensified the search for an ideal absorbable metallic stent material. Focus on Fe-based stents has been growing over the last decade, although the literature is limited to biocompatibility evaluation and clinical translation in *in vivo* animal studies. Our preclinical trial first proved the efficacy and safety of IBS in humans, and that this approach did not cause stent thrombosis, malposition, or restenosis. Furthermore, the nitride iron stent has many advantages, including good mechanical properties, elevated tensile strength, microhardness, radial strength, stiffness, and visibility under X-rays. Furthermore, our clinical trial attempted to investigate whether the degradation products of iron-based stents are toxic human organs. We measured the related indicators of iron metabolism before stenting because iron is a necessary element in the human body. In all cases, the indicators of iron metabolism were normal preoperatively. Since the other patients did not reach the 6-month follow-up period, iron metabolism was measured again in only three patients at 6-month follow-up; no significant abnormalities were found. Although the number of cases was small, our results are consistent with those arising from animal studies. For example, Lin et al. implanted 70 μm diameter nitride Fe stents into porcine coronary arteries for up to 53 months (31) and found that the corrosion products did not elicit toxic systemic effects on the heart, liver, spleen, lung, or kidney tissue.

Our clinical trial was also the first to investigate the improvements in right ventricular systolic and diastolic function after stenting. In patients with repaired CHD, residual lesions are common and can lead to impairment of right ventricular systolic and diastolic function. It is necessary to treat residual lesions because they are associated with a poorer prognosis (32, 33). The assessment of RV function by non-invasive methods is challenging because the right ventricle is dramatically modified by the surgical repair of CHD, with infundibular bulging and apical dilation and deformation, thus leading to a large range of RV shapes (34, 35), as well as contraction patterns and response to overload. With regards to systolic function, we measured TAPSE, pulsed Doppler S wave (S’) and FAC to assess RV function because this approach was both feasible and reproducible. We found that TAPSE correlated significantly with S’ (*p* < 0.05). Mean TAPSE and S’ values increased after relieving pressure overload, although there were no significant differences due to small samples. Several previous studies have reported similar results in patients who underwent cardiac surgery; for example, one study showed that the values of TAPSE and S’ were increased in volume overload and decreased in pressure overload (36). FAC is a simple measure of RV systolic function. We found that FAC was normal before and after stenting; after one-month post-stenting, the FAC had increased significantly when compared to that before stenting. This indicated that the RV systolic function had improved. FAC has been shown to correlate well with RVEF when measured by MRI in the general population (37). However, in patients with repaired CHD, the relevance of FAC to RVEF remains controversial (38). At present, there were no better parameters to assess RV systolic function, although real-time three-dimensional echocardiography and three-dimensional knowledge-based reconstruction can be used for the accurate estimation of RV volumes and RVEF by echocardiography in repaired CHD; this approach, however, requires further research.

To date, there is a lack of guidance for the assessment and quantification of RV diastolic function. However, many conditions have been shown to be associated with RV diastolic dysfunction, including CHD, cardiomyopathies, left-sided valvular heart diseases, systemic conditions and various vasculitides (12). Therefore, accurate assessment of RV diastolic function is also important. We attempted to evaluate RV diastolic function from different echocardiographic views and modalities (primarily 2-dimensional, PW, and tissue Doppler). One-month post-stenting, E′/A′ was significantly different from that before stenting; three months post-stenting, E/A was significantly different. These results demonstrated a slight change in RV diastolic function. This small change was related to our small number of cases and short follow-up time; furthermore, all patients still had tricuspid and pulmonary valve regurgitation. Severe tricuspid and pulmonary regurgitation are also an important prognostic factor affecting right ventricular diastolic function. Our clinical trial only solved the pressure load, but did not significantly improve the volume load, which requires further study.

### Study limitations

The prospective and multicenter clinical trial had some limitations. First, our follow-up period was two years, however, the preliminary trial only evaluated the efficacy and safety of stenting for branch pulmonary artery stenosis using a biodegradable nitride iron stent (IBS^®^ Angel™) and right ventricular systolic and diastolic function during the initial three months, the follow-up was too short since here after three months most of all the biodegradable stents were still there. Secondly, the relatively small size of the cohort may have biased the results. Finally, there was a lack of guidance for the assessment and quantification of RV function in congenital heart diseases in pediatric patients. Although MRI is the gold standard, it is still difficult to complete MRI examination for infants in China. The image quality of MRI is poor due to the long operation time, the difficulty for infants to cooperate and the need for sedation. 2D or 3D RV speckle-tracking echocardiology is the focus of future research. Clinical applications of strain imaging to assess systolic and diastolic function in children with complex congenital heart disease have recently been reported. However, the mean values and associated variations of these strain values have not to be firmly established in children, which limited the study.

## Conclusion

In this preclinical trial, we first evaluated the efficacy and safety of IBS for branch pulmonary artery stenosis in children. The short-term results indicated that the biodegradable Fe- based stent is the perfect choice for infants or children. The diameter of the lumen was significantly improved and no stent thrombosis, malposition, or restenosis were observed. Nevertheless, small improvements in RV systolic and diastolic function were noted after successful transcatheter intervention, although there were no significant differences due to the small sample size. We believe that a comprehensive evaluation is now necessary in patients with biodegradable Fe-based stents, including the assessment of long-term safety, exercise capacity and ventricular function. Importantly, not all patients with branch PAS benefit from endovascular stenting; further evaluation is desirable to understand and predict whether intervention is likely to be beneficial.

## Data availability statement

The original contributions presented in this study are included in the article/supplementary material, further inquiries can be directed to the corresponding author.

## Ethics statement

The studies involving human participants were reviewed and approved by the Clinical Research Ethic Committee of Guangdong Provincial People’s Hospital. Written informed consent to participate in this study was provided by the participants’ legal guardian/next of kin.

## Author contributions

LS and J-jL contributed to conception and manuscript writing. Y-kX, Y-mX, and S-sW contributed to design and formal analysis. Z-wZ contributed to editing, supervision, and funding acquisition. All authors contributed to manuscript revision, read, and approved the submitted version.
